# Psychological well-being in cognitively intact adults aged 80+: Findings from the SuperAging Research Initiative

**DOI:** 10.3389/fpsyg.2026.1819539

**Published:** 2026-08-26

**Authors:** Claire M. Alexander, Sophia A. Moore, Kylie R. Kadey, Ana W. Capuano, Elena Badillo Goicoechea, Rhiana Schafer, Victoria Balog, Felicia C. Goldstein, Ozioma C. Okonkwo, Angela C. Roberts, Adam Martersteck, SuperAging Research Initiative, Amanda Cook Maher, Emily J. Rogalski

**Affiliations:** 1 Psychiatry Department, University of Michigan, Ann Arbor, MI, United States; 2 VA Ann Arbor Healthcare System, Mental Health Service, Ann Arbor, MI, United States; 3 Healthy Aging and Alzheimer’s Research Care (HAARC) Center, Department of Neurology, University of Chicago, Chicago, IL, United States; 4 Goizuetta Alzheimer’s Disease Research Center, Department of Neurology, Emory University School of Medicine, Atlanta, GA, United States; 5 Wisconsin Alzheimer’s Disease Research Center and Department of Medicine, University of Wisconsin School of Medicine and Public Health, Madison, WI, United States; 6 Department of Computer Science, Western University, London, ON, Canada; 7 School of Communication Sciences and Disorders, Western University, London, ON, Canada; 8 Canadian Centre for Activity and Aging, London, ON, Canada

**Keywords:** aging, cognition, cognitive resilience, emotional well-being, exceptional aging, psychological well-being

## Abstract

Aspects of psychological well-being have shown positive relationships with cognitive aging and may support memory resilience. To explore this hypothesis, psychological well-being was assessed in SuperAgers (individuals with exceptional memory for age; *n* = 98) and cognitively-average-for-age Controls (“Controls,” *n* = 128), all aged 80+ and enrolled in the multisite, longitudinal SuperAging Research Initiative. Psychological well-being was measured using the NIH Toolbox®-Emotion Battery (NIHTB-EB). Emotional well-being was characterized in SuperAgers and Controls, and associations with cognitive group and demographic characteristics were examined using ordinary least squares regression models. Both SuperAgers and Controls reported generally favorable psychosocial profiles, with several individual NIHTB-EB dimension scores directionally favorable relative to the general adult reference mean. Within this cognitively intact cohort, SuperAger status was not associated with differences in NIHTB-EB summary scores or individual dimensions relative to Controls, suggesting that positive emotional functioning may be a shared feature of successful aging. Across participants, age showed modest associations with select social–emotional outcomes, including friendship and emotional support. This study extends prior characterization of the SuperAging phenotype and identifies generally favorable self-reported psychosocial functioning among cognitively intact and cognitively exceptional adults aged 80+, supporting psychosocial well-being as a potential common resilience factor in late life.

## 1 Introduction

Although most older adults experience some degree of cognitive change with increasing age, particularly in memory, there are consistent reports of an exceptional memory aging phenotype that demonstrates superior episodic memory performance beyond age 80 (Cook Maher et al., 2022; Gefen et al., 2021; Harrison et al., 2012; Rogalski et al., 2013). SuperAgers, defined as community-dwelling adults age 80 and older who perform at least as well as average 50–65-year-olds on episodic memory measures and in the at-least-average-for-age range in other cognitive domains (Harrison et al., 2012; Rogalski et al., 2013), represent this exceptional memory aging phenotype. SuperAgers and their cognitively average-for-age peers are prospectively enrolled in the multisite, longitudinal SuperAging Research Initiative, which aims to identify factors supporting memory resilience in late life. Prior studies of this cohort suggest that SuperAgers have unique bio-psychosocial characteristics that distinguish them from their cognitively-average, similarly-aged peers. For example, SuperAgers have shown structural and neuropathologic differences in brain regions associated with emotional and cognitive functioning, including greater cortical thickness in the anterior cingulate cortex (ACC) and lower neurofibrillary tangle burden in the ACC and entorhinal cortex on average (Gefen et al., 2021; Harrison et al., 2012; Rogalski et al., 2013). SuperAgers also report lifestyle and social features consistent with resilience, including regular physical activity and continued work beyond retirement (Burke et al., 2019). Furthermore, SuperAgers report higher quality interpersonal relationships compared to their cognitively average-for-age peers (Cook Maher et al., 2017) and, in an early investigation, most SuperAgers scored highly in extraversion on the NEO measure of personality (Rogalski et al., 2019). Qualitative reviews reveal psychological resilience and sustained engagement (e.g., volunteering, avid reading, travel) even among SuperAgers with histories of adversity or depression (Rogalski et al., 2019). Together, these observations motivate a more comprehensive, quantitative characterization of psychological well-being in SuperAgers using standardized measures. This approach may help determine whether psychosocial well-being differs between exceptional and average-for-age memory phenotypes and contextualize these findings within broader patterns of late-life resilience.

Studies outside of the SuperAging Research Initiative have examined psychological characteristics of cognitively normal older adults (typically conceptualized as age 65+) and the “oldest old,” often defined as individuals over 80 or 85 years. Across cohorts, older adults frequently report higher life satisfaction compared to middle-aged adults, as well as lower perceived stress and aspects of negative affect compared to general population norms (Hajek and König, 2022; Lee et al., 2020; Mather et al., 2024). In the Assessing Reliable Measurement in Alzheimer’s Disease and cognitive Aging (ARMADA) study, which aimed to validate modules from the NIH Toolbox® (NIHTB), cognitively normal adults age 65+ reported slightly lower-than-average perceived stress and aspects of negative affect on the NIHTB-Emotion Battery, however, there were no significant differences between NIHTB reports from “older adults” (age 65–84) and the “oldest old” (age 85+) (Mather et al., 2024). Positive psychological factors, including life satisfaction, have been associated with more social relationships and a larger social network, lower functional impairment, and better self-rated health (Hajek and König, 2022; Sulandari et al., 2025).

Other studies have linked psychological well-being, social factors, and cognitive functioning among middle-aged and older adults. Psychological well-being (including aspects of autonomy, self-realization, and pleasure) has been associated with global cognitive functioning and episodic memory in a population-based study cross-sectionally (Llewellyn et al., 2008). A large, longitudinal study using data from the English Longitudinal Study of Ageing (ELSA) has also demonstrated a positive connection between psychological well-being and cognition, including verbal memory specifically, even after controlling for age and depression (Allerhand et al., 2014). Additionally, perceptions of social support appear positively related to cross-sectional and longitudinal verbal memory performance among middle-aged, older, and oldest old adults (Ge et al., 2017; Zhai et al., 2024; Zuelsdorff et al., 2019). In light of these findings, it appears that better social support and other aspects of psychological well-being are associated with unimpaired memory skills in aging and may contribute to exceptional aging. Age and sex/gender may moderate these findings, with some analyses showing the strongest positive relationship between social engagement and cognition among the oldest old (Zhai et al., 2024), and others demonstrating a positive relationship between social support and cognition only among women (Joyce et al., 2021). Additional studies, including a meta-analysis, have found sex differences in psychological well-being in older adulthood, with men reporting higher subjective well-being, self-acceptance, and purpose in life than women (Carmel, 2019; Pinquart and Sörensen, 2001). However, some analyses find that cognitively intact older women report greater emotional support and group involvement compared to cognitively intact older men who report more social ties but also more negative interactions (Seeman et al., 2001). Given these findings, further characterization of psychosocial well-being in cognitively intact individuals age 80+, and how both demographics and cognitive phenotype may relate to these features, is warranted and may inform hypotheses about resilience in late life.

The current study builds on early investigations of psychological well-being in the Chicagoland SuperAging cohort (Cook Maher et al., 2017) by using the NIH Toolbox® Emotion Battery (NIHTB-EB) to characterize psychosocial functioning in a larger, more geographically and racially diverse sample. The goal of these analyses is to (1) characterize emotional well-being in cognitively unimpaired 80+ −year-olds and (2) determine whether SuperAgers differ from their cognitively average peers (Controls). Based on prior research, it was hypothesized that SuperAgers would report higher levels of positive affect, life satisfaction, emotional support, friendship, social satisfaction, and psychological well-being; and lower levels of perceived stress and negative affect, compared to the cognitively average-for-age sample and relative NIH Toolbox® normative values.

## 2 Methods

### 2.1 Participants

Data were obtained from the SuperAging Research Initiative, a multisite longitudinal study of English-speaking, community-dwelling adults aged ≥80 years without neurologic disorders known to affect cognition and without significant uncontrolled medical conditions at enrollment. The Initiative enrolls participants across five North American sites (Ann Arbor/Detroit, MI; Atlanta, GA; Chicago, IL; London, ON, Canada; and Madison, WI) (Rogalski et al., 2024). Recruitment into the SuperAging Research Initiative uses multi-pronged, community-engaged research strategies, implemented in partnership with local organizations and community stakeholders, including community-based outreach and educational events, outreach through Alzheimer’s Disease Research Centers and affiliated clinics, earned media and print/digital advertisements, participant referrals (“word of mouth”), and registry/interest-list outreach where applicable. Recruitment activities emphasized trust-building, transparency, and accessibility, with materials tailored for older adults and opportunities for bidirectional questions and feedback.

Participants were classified as SuperAgers or Controls based on prospectively specified cognitive performance criteria; all classifications underwent case-by-case adjudication by two authors (ER, ACM) to ensure consistent application of criteria (Rogalski et al., 2024; Harrison et al., 2012; Rogalski et al., 2013). Briefly, SuperAgers were required to perform at least as well as middle-aged controls on episodic memory testing (Rey Auditory Verbal Learning Test delayed-recall raw score ≥9; Schmidt, 2004) and in the average range or higher for their age on tests in non-memory domains (30-item Boston Naming Test; Trail Making Test Part B; and Category Fluency Test). Please see prior literature by Rogalski et al. (2013), Rogalski et al. (2024) and Harrison et al. (2012) for complete details. Controls were required to perform in the average range for their age on memory testing, and at least average for their age in non-memory domains. At the time of analyses, 226 participants met criteria, including 128 Controls and 98 SuperAgers. All study procedures were approved by the appropriate Institutional Review Boards of participating sites, and written informed consent was obtained from all participants in accordance with the Declaration of Helsinki.

#### 2.2 Demographics

Participants self-reported their age, sex, race/ethnicity, years of education, living status, and marital status. Similar to previous SuperAging studies, self-reported sex differed between SuperAgers and Controls, with the SuperAger group showing a higher proportion of females (74.5%, compared to 50.8% female for cognitively-average Controls, *p* < 0.001); as such, models included sex as a variable. Median age, mean years of education, and proportion of racial/ethnic identity did not differ between SuperAgers and Controls (*p* > 0.05).

#### 2.3 NIH ToolBox®-Emotion Battery (NIHTB-EB)

Participants completed self-report inventories of psychological functioning measures from the NIHTB-EB, a tool for brief computerized assessment across cognition, emotion, sensation, and motor functioning (Gershon et al., 2013). The NIHTB-EB offers a wide array of dimensions across psychological well-being, social relationships, stress and self-efficacy, and negative affect (see Supplementary Table S1), which are either computer-adaptive or fixed-form surveys. The NIHTB-EB has been well-validated in healthy and neurologic samples (Babakhanyan et al., 2018) and has been shown to be broadly stable across age groups (Mather et al., 2024). Initial validation of the NIHTB-EB (Salsman et al., 2013) grouped these dimensions into domains of Psychological Well-Being, Social Relationships, Stress and Self-Efficacy, and Negative Affect (as delineated in Supplementary Table S1), based on literature review and expert input. A later factor analysis by Babakhanyan et al. (2018) showed that a three-factor structure best suited data for both English- and Spanish-speaking normative cohorts (also seen in Supplementary Table S1), and this factor structure has been used in several lines of research examining people with neurologic injury and HIV, respectively (Babakhanyan et al., 2019; Brody et al., 2023). These factors have also been referred to as “subdomains” or “summary scores” (Ingram and Karr, 2024). In this study, the Babakhanyan et al. (2018) three-factor structure was applied, referred to as “summary scores” for consistency, of Psychological Well-Being, Social Satisfaction, and Negative Affect.

NIHTB-EB translates raw scores for non-computer adaptive measures and theta scores for computer adaptive measures into “uncorrected” T-scores based on the general population of English-speaking adults ages 18–85 living in the United States (Casaletto et al., 2015). T-scores have a mean of 50 and a standard deviation of 10. Higher T-scores indicate that participants report greater levels of the measured trait. Scores at least one standard deviation from the mean in the unfavorable direction for a given scale have been described as “potentially problematic,” rather than abnormal, and should be interpreted in the context of other clinical and psychosocial information (Babakhanyan et al., 2018; Ingram and Karr, 2024). Because the present cohort included adults aged 80–100 years, T-scores were used descriptively to contextualize individual NIHTB-EB dimension scores relative to the general adult reference mean; these comparisons were not formal tests against an age-matched normative sample.

To derive the NIHTB-EB summary (composite) scores following Babakhanyan et al. (2018), a three-factor confirmatory factor analysis (CFA) was specified using the lavaan R package v0.6–20 (Rosseel et al., 2025) with robust maximum likelihood estimation (MLR) and full-information maximum likelihood for missing data. Factors were allowed to correlate and were defined as: (1) Negative Affect (Anger Affect, Anger Hostility, Sadness, Fear Affect, Perceived Stress), (2) Social Satisfaction (Friendship, Emotional Support, Instrumental Support, Loneliness, Perceived Rejection), and (3) Psychological Well-Being (Life Satisfaction, Meaning/Purpose, Positive Affect). For the analytical sample in this study (*n* = 226) the three-factor model had acceptable fit indices: robust *χ*^2^(62) = 156.80, *p* < 0.001; CFIᵣ = 0.931; TLIᵣ = 0.913; RMSEAᵣ = 0.085 (90% CI [0.068, 0.102]); SRMR = 0.049. Results had expected directions and magnitudes (Negative Affect: 0.47–0.85; Social Satisfaction: 0.53–0.85 in absolute value, with Loneliness = −0.85 and Perceived Rejection = −0.65 indicating reverse scoring; Psychological Well-Being: 0.67–0.73), and latent factors were strongly interrelated (*r* = −0.79 to 0.82; all *p* < 0.001). Following the normative paper, we computed summary scores by weighting IRT theta scores with the CFA standardized loadings within each larger summary domain and rescaling to T-scores (M = 50, SD = 10); negative-valence indicators in Social Satisfaction were reversed before aggregation. Formulas for composite computation are presented alongside those from Babakhanyan et al. (2018) in Supplementary Table S2 to document the equivalence of the scoring pipeline.

#### 2.4 Statistical analyses

Initial analyses examined the distribution of the variables. For continuous variables, analyses examined if there were strong departures from normality using a combination of normality tests and visual inspection of the distributions. Demographic variables were characterized by cognitive group as seen in Table 1. Age, sex, race, education, and Wechsler Test of Adult Reading (WTAR) scores were used to account for the known effects of demographics on our variables of interest, to account for differences in proportions of sex between cognitive groups, and to account for potential effects of formal education. Age, education, and WTAR score were median-centered for subsequent analyses. NIHTB-EB dimensions were characterized by cognitive group using descriptives and *t*-tests.

**Table 1:** Demographics.

Demographic variable	SuperAgers (*n* = 98)	Cognitively-average controls (*n* = 128)	Full sample (*n* = 226)
Median age (Q1, Q3), [min–max]	83 (81, 86), [80–100]	84 (81, 87), [80–100]	84 (81, 87), [80–100]
Sex: % (*N*)			
Male	25.5% (25)	49.2% (63)	38.9% (88)
Female	74.5% (73)	50.8% (65)	61.1% (138)
Education in years (mean, SD)	17.11 (2.32), [12–20]	16.95 (2.39), [11–20]	17.02 (2.35), [11–20]
Race: % (*N*)			
White	83.7% (82)	78.9% (101)	81.0% (183)
Black/African American	16.3% (16)	20.3% (26)	18.6% (42)
Asian	—	0.8% (1)	0.4% (1)
Marital status: % (*N*)			
Married	38.1% (37)	42.9% (54)	40.8% (91)
Widowed	35.1% (34)	33.3% (42)	34.1% (76)
Divorced	17.5% (17)	15.9% (20)	17.0% (37)
Separated	—	0.8% (1)	0.4% (1)
Never married	6.2% (6)	4.8% (6)	5.4% (12)
Domestic partnership	3.1% (3)	2.3% (3)	2.7% (6)
Living situation: % (*N*)			
Alone	47.4% (46)	50.4% (63)	49.1% (109)
With one person: partner/spouse	41.2% (40)	44.8% (56)	43.2% (96)
With one person: non-partner/spouse	6.2% (6)	4.0% (5)	5.0% (11)
With group, in private residence	3.1% (3)	0.8% (1)	1.8% (4)
In a group home	2.0% (2)	—	0.9% (2)

Missing/unknown: *n* = 3 for marital status; *n* = 4 for living situation.

Based on prior research in the SuperAging and ARMADA studies, potential phenotypic group differences in the individual NIHTB-EB dimensions, with outcomes of Positive Affect, Life Satisfaction, Emotional Support, Friendship, and Perceived Stress, were examined using a series of ordinary least squares regressions. Model 1 included age (years) and sex (female = 1, male = 0). Model 2 added cognitive group (SuperAger = 1, Control = 0). Model 3 added race (White = 0, non-White = 1), years of education, and WTAR age-corrected score. Model 4 added the sex-by-phenotypic group interaction. *R*^2^ and adjusted *R*^2^ are reported. Model assumptions were checked and found to be acceptable.

### 3 Results

Descriptive demographics for SuperAgers and Controls are provided in Table 1. The median age of both groups was in the lower/mid-80s (range 80–100) and included primarily White and Black individuals (81% and 18.6% respectively), mostly married or widowed (40.8% and 34.1% respectively), with about half of the participants living alone.

Descriptive statistics for NIHTB-EB dimensions and groupwise difference tests between SuperAgers and Controls are shown in Table 2. For several dimensions, both SuperAger and Control group means were >0.5 standard deviations from the general population mean. This includes both groups demonstrating slightly higher General Life Satisfaction, lower Perceived Stress, and lower Anger (Affect, Physical Aggression, and Hostility) compared to the general population. Both groups also showed scores at approximately 0.5 standard deviations below the population mean for Sadness. Unadjusted groupwise tests did not indicate differences between SuperAgers and Controls on any NIHTB-EB dimension (*p* > 0.05) (see Table 2).

**Table 2:** Descriptives and group comparisons for NIHTB-EB T-scores by cognitive group.

NIHTB-EB dimension	SuperAgers		Cognitively-average controls		Group difference test	
	Mean	SD	Mean	SD	*t*	*p*
Positive affect	49.95	7.08	50.20	7.02	−0.26	0.795
General life satisfaction	58.17	8.42	56.87	9.48	1.09	0.275
Meaning and purpose	51.62	9.02	51.70	8.79	−0.07	0.946
Emotional support	47.63	8.65	47.72	8.05	−0.08	0.939
Instrumental support	50.34	9.24	50.58	9.33	−0.19	0.849
Friendship	51.48	8.79	52.21	7.84	−0.65	0.517
Loneliness	49.14	8.73	48.43	8.70	0.61	0.543
Perceived rejection	47.21	8.20	47.45	8.44	−0.21	0.836
Perceived hostility	46.19	7.38	47.51	7.67	−1.30	0.194
Self-efficacy	53.22	8.09	53.26	8.12	−0.03	0.976
Perceived stress	43.26	8.72	44.51	8.61	−1.08	0.283
Fear-affect	46.68	8.89	48.41	8.56	−1.47	0.144
Fear somatic arousal	48.53	7.95	48.14	8.98	0.35	0.730
Sadness	44.37	8.66	45.39	9.02	−0.86	0.388
Anger-affect	43.84	7.98	44.91	8.26	−0.99	0.323
Anger-hostility	41.38	6.61	42.23	7.61	−0.90	0.367
Anger-physical aggression	44.43	5.44	44.16	5.43	0.37	0.709

*NIHTB-EB: NIH toolbox®-Emotion Battery. SuperAgers *n* = 98, Cognitively-average controls *n* = 128. Degrees of freedom vary by scale due to missing data.

Linear regressions were used to model the adjusted associations of cognitive group and demographic characteristics on aspects of emotional well-being, including NIHTB-EB summary scores and selected individual dimensions (Supplementary Tables S3–S10). In models including demographics terms (Model 1 for each outcome), older age was associated with lower Social Satisfaction (*p* = 0.031), Friendship (*p* = 0.017), General Life Satisfaction (*p* = 0.034), and Emotional Support (*p* = 0.020) as well as a trend-level association with lower Psychological Well-Being (Supplementary Tables S4–S6, S8, S9). Age was not associated with perceived stress. While sex was included as a covariate in all models and was not related to any NIHTB-EB outcome, the interpretation of sex effects should be made cautiously given the relatively small number of men in the sample, particularly in the SuperAger group (Table 1). Models then tested if SuperAgers and Controls differed on NIHTB-EB outcomes, adjusting for demographics (Model 2 for each outcome; regression coefficients for SuperAging status are shown in Table 3. Across these models including cognitive group and demographic covariates, SuperAger status was not associated with any NIHTB-EB summary score or individual dimension. In Model 3, which additionally included race, education, and WTAR score, non-White race was associated with lower General Life Satisfaction (*p* = 0.005). In Model 4, sex × cognitive group interactions were not significant for any outcome (Supplementary Tables S3–S10).

**Table 3:** SuperAging status coefficients by outcome (regression).

Outcome	B for SuperAger status	SE	*t*	*p*
Negative affect	−1.71	1.11	−1.54	0.12
Social satisfaction	−0.44	1.17	−0.37	0.71
Psychological well-being	−0.12	1.06	−0.11	0.91
Positive affect	−0.35	0.98	−0.36	0.72
General life satisfaction	0.96	1.25	0.77	0.44
Emotional support	−0.28	1.15	−0.24	0.81
Friendship	−1.41	1.13	−1.24	0.22
Perceived stress	−1.44	1.20	−1.20	0.23

Values are regression coefficients for SuperAger status from Model 2 of separate ordinary least squares regression models. Each model included age, sex, and SuperAger status. SuperAger status was coded 1 = SuperAger and 0 = cognitively average-for-age Control; thus, negative coefficients indicate lower scores among SuperAgers relative to Controls after adjustment for age and sex. Negative Affect, Social Satisfaction, and Psychological Well-Being are summary scores; remaining outcomes are individual NIH Toolbox® Emotion Battery dimensions.

### 4 Discussion

This study extends the characterization of emotional and psychological functioning of SuperAgers as well as their cognitively average-for-age, similarly aged peers. Using factor-based NIHTB-EB summary scores as proposed by Babakhanyan et al. (2018), both SuperAgers and their cognitively average peers showed directionally favorable patterns on several individual NIHTB-EB dimensions relative to the general adult reference mean, including life satisfaction, perceived stress, anger, and sadness. These patterns align with broader evidence that psychological well-being and social connectedness are linked to healthier aging outcomes (Llewellyn et al., 2008). Within this cognitively intact 80+ cohort, SuperAger status was not associated with broad differences in NIHTB-EB dimensions or summary scores, and sex did not moderate these associations. Instead, chronological age showed modest associations with select social–emotional facets, including Friendship and Emotional Support, suggesting that age-related changes in social experience may be more salient than exceptional memory status for self-reported emotional functioning in late life. These age patterns are consistent with work showing reductions in the availability of social contacts with increasing age (Cornwell, 2011) and elevated risk for social isolation and loneliness in older adulthood (Donovan and Blazer, 2020). Measurement timing is important, as prior work in successful aging samples has linked higher emotional support at younger baseline ages (e.g., average ~mid-70s) to cognitive trajectories over time (Seeman et al., 2001), whereas the current cohort averaged ~85 years with a span up to 100. Future longitudinal analyses within the SuperAging Research Initiative have the potential to help clarify how socioemotional factors relate to cognitive trajectories in very late life.

Mean NIHTB-EB scores in both SuperAgers and Controls indicated higher life satisfaction along with lower perceived stress, sadness, and anger compared to general U.S. population norms. The generally positive emotional well-being reported by our sample is similar to the socioemotional outcomes of the oldest old of the ARMADA study (Mather et al., 2024). Prior literature has linked higher psychological well-being with better cognitive functioning in middle-aged and older adults (Llewellyn et al., 2008). Similarly, purpose in life has been associated with better cognitive functioning, though operationalization varied from lower risk for cognitive impairment (Boyle et al., 2010) to higher cognitive performances among a large sample of adults age 70+ without inclusion criteria related to cognitive status (Windsor et al., 2015). The restricted range of cognitive functioning and age in the present sample, comprised of cognitively normal and exceptional adults over age 80, may partly explain why associations observed in broader populations were attenuated here.

Social factors have also been related to positive cognitive outcomes in aging. For example, extraversion has been associated with lower risk of mild cognitive impairment (Schaeffer et al., 2024), which aligns with prior work showing that SuperAgers report higher positive relations with others (Cook Maher et al., 2017). Social well-being showed modest age-related variation with increasing participant age beyond age 80 across both cognitive groups, as seen in Friendship and Emotional Support. Thus, while social factors may contribute to a positive aging trajectory, this cognitively intact cohort also showed lower perceived social connection with increasing age.

Life satisfaction and positive affect were not associated with SuperAging status or age in the current analyses. Prior studies regarding age-related patterns of life satisfaction are mixed and may reflect cohort effects, cultural context, and unmeasured moderators (Cho et al., 2013; Sulandari et al., 2025). For example, a recent cross-sectional study recruiting across the lifespan indicated that life satisfaction increased with participants’ reported age, including after age 80, for British participants; however, older Indonesian participants indicated lower life satisfaction compared to their midlife peers (Sulandari et al., 2025). Another study demonstrated higher positive affect among octogenarians compared to centenarians, though predictors of positive affect differed by age: for octogenarians, positive affect was predicted by physical health and learning experiences, whereas for centenarians, positive affect was predicted by cognitive and physical functioning (Cho et al., 2013). The SuperAging Research Initiative enrolls individuals based on cognitive phenotype, irrespective of physical functioning or health beyond relative stability of chronic conditions and thus participants have a range of health conditions and physical functioning. Future studies may wish to explore the physical functioning and activity of SuperAgers to more thoroughly investigate its possible impact on cognitive and emotional resilience.

In this study, race (coded as non-White and White) was associated with General Life Satisfaction in the fully adjusted model, with non-White race associated with lower life satisfaction. This association should be interpreted cautiously. The binary race variable combined participants from heterogeneous racial and ethnic backgrounds, and the study did not measure socioeconomic resources, discrimination, social support, coping, or other social determinants that may contribute to variation in life satisfaction. Future studies of exceptional memory in aging would benefit from more comprehensive and appropriately granular assessment of these factors.

Several limitations to these findings exist related to the nature of our sample and methodology. First, there is a restricted range of NIHTB-EB scores in this sample, possibly limiting the sensitivity to detect smaller between-group differences in emotional well-being. Second, both groups comprised older adults without cognitive impairment who volunteered for a research study of exceptional late-life memory; this may yield more similar psychosocial profiles than would be expected relative to a general population sample of adults aged 80+. Generalizability of these findings are limited, as SuperAgers are a rare cognitive phenotype, though cognitively average Controls are recruited in a similar manner to understand potential differences between individuals with very strong memory and average-for-age memory. Third, given the sample size and identified effect sizes, nonsignificant findings should be interpreted with appropriate caution, as they may reflect limited statistical power rather than a true absence of association. Replication with larger samples is warranted as our ongoing recruitment of SuperAgers and controls continues. Social desirability bias may influence the reporting of psychosocial outcomes in general; however, all comparisons between cognitive groups as well as previously published NIHTB normative data used equivalent measures. Finally, interpretation of individual NIHTB-EB T-scores relative to population reference values warrants caution because the available reference sample spans adulthood through age 85 and does not fully age-match the present cohort, which included participants up to age 100.

In sum, SuperAgers as well as cognitively average-for-age controls showed directionally favorable self-reported psychosocial profiles on selected individual NIHTB-EB dimensions relative to the general adult reference mean, with modest age-related variation in select social–emotional facets. These findings extend psychosocial characterization of the SuperAging phenotype and highlight positive emotional functioning as a potential shared resilience feature in late life.

## Data Availability

The datasets presented in this article are not readily available because a request must be formally made for collaboration. Requests to access the datasets should be directed to https://haarc.center.uchicago.edu/collaborate/.

## Author disclaimer

This work was authored as part of the Contributor’s official duties as an Employee of the United States Government and is therefore a work of the United States Government. In accordance with 17 U.S.C. 105, no copyright protection is available for such works under U.S. Law.
