# Supplementary material for: Psychological well-being in cognitively intact adults aged 80+: Findings from the SuperAging Research Initiative
Source: Front Psychol. 2026 Aug 26;17:1819539. doi: 10.3389/fpsyg.2026.1819539 (PMC13553414; doi:10.3389/fpsyg.2026.1819539)
Supplement: Supplementary file 1 [file Table_1.docx]

**Supplemental Table 1. NIHTB-EB domains and dimensions.**

| **Domain** | **Babakhanyan et al., 2018** | **NIHTB Manual; Salsman et al., 2013** |
| --- | --- | --- |
| Psychological Well-Being | Positive affect*  General life satisfaction*  Meaning & purpose* | Positive affect*  General life satisfaction*  Meaning & purpose* |
| Social Satisfaction/  Social Relationships | Emotional support  Instrumental support  Loneliness (rev)  Friendship  Perceived rejection (rev) | Emotional support  Instrumental support  Loneliness  Friendship  Perceived hostility  Perceived rejection |
| Stress and Self-Efficacy | N/A | Perceived stress  Self-efficacy* |
| Negative Affect | Anger-affect*  Anger-hostility  Fear-affect*  Perceived stress  Sadness* | Anger-affect*  Anger-hostility  Anger-physical aggression  Fear-affect*  Fear-somatic arousal  Sadness* |
| Supplemental | Self-efficacy*  Perceived hostility  Fear-somatic arousal  Anger-physical aggression | N/A |

*Notes.* *denotes computer-adaptive tests, as opposed to fixed form tests; computer-adaptive measures give outcomes as theta scores, which are then converted to T-scores, whereas fixed-form tests use raw scores that are converted to T-scores. Note that Loneliness and Perceived Rejection are reverse coded when used for summary scoring for Social Satisfaction.

**Supplemental Table 2. NIHTB-EB composite formulas from CFA.**

| Factor | Sample | Formula | Mean | SD |
| --- | --- | --- | --- | --- |
| Negative Affect | SuperAgers & Cognitively-Average Controls | ((((((a×0.722)+(b×0.467)+(c×0.839)+(d×0.820)+(e×0.847))/5]-(-0.349))/0.444)×10+50 | -0.349 | 0.44 |
|  | Babakhanyan | ((((a×0.774)+(b×0.644)+(c×0.842)+(d×0.827)+(e×0.835))/5]-(-0.02))/0.58)×10+50 | -0.02 | 0.58 |
| Social Satisfaction | SuperAgers & Cognitively-Average Controls | ((((((i×0.733)+((j×-1)×0.701)+(k×0.533)+((l×-1)×-0.852)+((m×-1)×-0.650))/5]-(0.014))/0.202)×10+50 | 0.014 | 0.20 |
|  | Babakhanyan | ((((i×0.709)+((j×-1)×0.804)+(k×0.760)+((l×0.586)+((m×-1)×0.703))/5]-(0.03))/0.53)×10+50 | 0.03 | 0.53 |
| Psychological Well-Being | SuperAgers & Cognitively-Average Controls | ((((((f×0.669)+(g×0.718)+(h×0.731))/3]-(0.244))/0.463)×10+50 | 0.244 | 0.46 |
|  | Babakhanyan | ((((f×0.752)+(g×0.729)+(h×0.879))/3]-(0.07))/0.67)×10+50 | 0.07 | 0.67 |

*Note. N*=226 for SuperAgers & cognitively-average Controls in the present study; *N*=1,036 for Babakhanyan et al., 2018.

**Supplemental Table 3. Negative Affect (Summary Score) Regression.**

|  | B | SE (B) | R square | R square (adj.) | F | df1, df2 | t | *p* |
| --- | --- | --- | --- | --- | --- | --- | --- | --- |
| Model 1 |  |  | 0.003 | -0.006 | 0.31 | 2, 223 |  | 0.75 |
| Age | 0.04 | 0.13 |  |  |  |  | 0.32 | 0.75 |
| Sex | 0.78 | 1.09 |  |  |  |  | 0.71 | 0.48 |
| Model 2 |  |  | 0.013 | 0.000 | 0.99 | 3, 222 |  | 0.40 |
| SuperAger Status | -1.71 | 1.11 |  |  |  |  | -1.54 | 0.13 |
| Model 3 |  |  | 0.012 | -0.016 | 0.42 | 6, 212 |  | 0.86 |
| Education | -0.07 | 0.24 |  |  |  |  | -0.29 | 0.77 |
| WTAR (SS) | -0.00 | 0.01 |  |  |  |  | -0.14 | 0.89 |
| Race | 0.32 | 1.40 |  |  |  |  | 0.23 | 0.82 |
| Model 4 |  |  | 0.012 | -0.021 | 0.36 | 7, 211 |  | 0.92 |
| SuperAger Status*Sex | 0.07 | 2.39 |  |  |  |  | 0.030 | 0.98 |

*Note.* *N*=226. SuperAger status was coded as 1=SuperAger, 0=Cognitively Average Control. WTAR: Wechsler Test of Adult Reading, SS: standardized score, adj.: adjusted. Race was coded as White (0) or non-White (1). Sex was coded as male (0) or female (1). SuperAger Status*Sex: interaction of SuperAger status and self-reported sex. Continuous predictors centered at their median: age 84, education 18 and WTAR 118. **Bolded models and predictors** indicate statistical significance.

**Supplemental Table 4. Social Satisfaction (Summary Score) Regression.**

|  | B | SE (B) | R square | R square (adj.) | F | df1, df2 | t | *p* |
| --- | --- | --- | --- | --- | --- | --- | --- | --- |
| **Model 1** |  |  | **0.027** | **0.018** | **3.05** | **2, 222** |  | **0.049** |
| **Age** | **-0.29** | **0.13** |  |  |  |  | **-2.17** | **0.031** |
| Sex | -1.52 | 1.15 |  |  |  |  | -1.32 | 0.19 |
| Model 2 |  |  | 0.027 | 0.014 | 1.80 | 3, 221 |  | 0.11 |
| SuperAger Status | -0.44 | 1.17 |  |  |  |  | -0.37 | 0.71 |
| Model 3 |  |  | 0.026 | -0.002 | 0.99 | 6, 211 |  | 0.47 |
| Education | 0.00 | 0.25 |  |  |  |  | 0.01 | 0.99 |
| WTAR (SS) | -0.01 | 0.01 |  |  |  |  | -0.61 | 0.54 |
| Race | -0.03 | 1.47 |  |  |  |  | -0.02 | 0.98 |
| Model 4 |  |  | 0.026 | -0.006 | 0.88 | 7, 210 |  | 0.58 |
| SuperAger Status*Sex | 0.75 | 2.49 |  |  |  |  | 0.30 | 0.76 |

*Note.* *N*=226. SuperAger status was coded as 1=SuperAger, 0=Cognitively Average Control. WTAR: Wechsler Test of Adult Reading, SS: standardized score, adj.: adjusted. Race was coded as White (0) or non-White (1). Sex was coded as male (0) or female (1). SuperAger Status*Sex: interaction of SuperAger status and self-reported sex. Continuous predictors centered at their median: age 84, education 18 and WTAR 118. **Bolded models and predictors** indicate statistical significance.

**Supplemental Table 5. Psychological Well-Being (Summary Score) Regression.**

|  | B | SE (B) | R square | R square (adj.) | F | df1, df2 | t | *p* |
| --- | --- | --- | --- | --- | --- | --- | --- | --- |
| Model 1 |  |  | 0.019 | 0.010 | 1.31 | 2, 223 |  | 0.12 |
| Age | -0.23 | 0.12 |  |  |  |  | -1.92 | 0.056 |
| Sex | 0.69 | 1.04 |  |  |  |  | 0.67 | 0.51 |
| Model 2 |  |  | 0.019 | 0.006 | 0.89 | 3, 222 |  | 0.23 |
| SuperAger Status | -0.12 | 1.06 |  |  |  |  | -0.11 | 0.91 |
| Model 3 |  |  | 0.020 | -0.008 | 0.58 | 6, 212 |  | 0.63 |
| Education | 0.04 | 0.23 |  |  |  |  | 0.18 | 0.86 |
| WTAR (SS) | -0.00 | 0.01 |  |  |  |  | -0.41 | 0.68 |
| Race | -0.66 | 1.33 |  |  |  |  | -0.49 | 0.62 |
| Model 4 |  |  | 0.026 | -0.006 | 0.72 | 7, 211 |  | 0.58 |
| SuperAger Status*Sex | 2.63 | 2.26 |  |  |  |  | 1.16 | 0.25 |

*Note.* *N*=226. SuperAger status was coded as 1=SuperAger, 0=Cognitively Average Control. WTAR: Wechsler Test of Adult Reading, SS: standardized score, adj.: adjusted. Race was coded as White (0) or non-White (1). Sex was coded as male (0) or female (1). SuperAger Status*Sex: interaction of SuperAger status and self-reported sex. Continuous predictors centered at their median: age 84, education 18 and WTAR 118. **Bolded models and predictors** indicate statistical significance.

**Supplemental Table 6. Friendship Regression.**

|  | B | SE (B) | R square | R square (adj.) | F | df1, df2 | t | *p* |
| --- | --- | --- | --- | --- | --- | --- | --- | --- |
| **Model 1** |  |  | **0.032** | **0.023** | **3.65** | **2, 223** |  | **0.028** |
| **Age** | **-0.31** | **0.13** |  |  |  |  | **-2.40** | **0.017** |
| Sex | 1.20 | 1.11 |  |  |  |  | 1.08 | 0.28 |
| **Model 2** |  |  | **0.038** | **0.025** | **2.95** | **3, 222** |  | **0.033** |
| SuperAger Status | -1.41 | 1.13 |  |  |  |  | -1.24 | 0.22 |
| Model 3 |  |  | 0.042 | 0.015 | 1.49 | 6, 212 |  | 0.16 |
| Education | -0.11 | 0.24 |  |  |  |  | -0.46 | 0.65 |
| WTAR (SS) | -0.00 | 0.01 |  |  |  |  | -0.51 | 0.61 |
| Race | 0.02 | 1.42 |  |  |  |  | 0.01 | 0.99 |
| Model 4 |  |  | 0.042 | 0.010 | 1.27 | 7, 211 |  | 0.24 |
| SuperAger Status*Sex | 0.30 | 2.42 |  |  |  |  | 0.13 | 0.90 |

*Note.* *N*=226. SuperAger status was coded as 1=SuperAger, 0=Cognitively Average Control. WTAR: Wechsler Test of Adult Reading, SS: standardized score, adj.: adjusted. Race was coded as White (0) or non-White (1). Sex was coded as male (0) or female (1). SuperAger Status*Sex: interaction of SuperAger status and self-reported sex. Continuous predictors centered at their median: age 84, education 18 and WTAR 118. **Bolded models and predictors** indicate statistical significance.

**Supplemental Table 7. Positive Affect Regression.**

|  | B | SE (B) | R square | R square (adj.) | F | df1, df2 | t | *p* |
| --- | --- | --- | --- | --- | --- | --- | --- | --- |
| Model 1 |  |  | 0.005 | -0.004 | 0.58 | 2, 223 |  | 0.56 |
| Age | -0.12 | 0.11 |  |  |  |  | -1.07 | 0.29 |
| Sex | -0.14 | 0.96 |  |  |  |  | -0.15 | 0.88 |
| Model 2 |  |  | 0.006 | -0.008 | 0.43 | 3, 222 |  | 0.73 |
| SuperAger Status | -0.35 | 0.98 |  |  |  |  | -0.36 | 0.72 |
| Model 3 |  |  | 0.014 | -0.014 | 0.49 | 6, 212 |  | 0.81 |
| Education | -0.19 | 0.21 |  |  |  |  | -0.90 | 0.37 |
| WTAR (SS) | -0.00 | 0.01 |  |  |  |  | -0.55 | 0.58 |
| Race | 0.81 | 1.22 |  |  |  |  | 0.66 | 0.51 |
| Model 4 |  |  | 0.019 | -0.013 | 0.60 | 7,211 |  | 0.76 |
| SuperAger Status*Sex | 2.28 | 2.08 |  |  |  |  | 1.10 | 0.27 |

*Note.* *N*=226. SuperAger status was coded as 1=SuperAger, 0=Cognitively Average Control. WTAR: Wechsler Test of Adult Reading, SS: standardized score, adj.: adjusted. Race was coded as White (0) or non-White (1). Sex was coded as male (0) or female (1). SuperAger Status*Sex: interaction of SuperAger status and self-reported sex. Continuous predictors centered at their median: age 84, education 18 and WTAR 118. **Bolded models and predictors** indicate statistical significance.

**Supplemental Table 8. General Life Satisfaction Regression.**

|  | B | SE (B) | R square | R square (adj.) | F | df1, df2 | t | *p* |
| --- | --- | --- | --- | --- | --- | --- | --- | --- |
| Model 1 |  |  | 0.021 | 0.012 | 2.42 | 2, 223 |  | 0.09 |
| **Age** | **-0.31** | **0.14** |  |  |  |  | **-2.13** | **0.034** |
| Sex | 0.51 | 1.23 |  |  |  |  | 0.41 | 0.68 |
| Model 2 |  |  | 0.024 | 0.011 | 1.80 | 3, 222 |  | 0.15 |
| SuperAger Status | 0.96 | 1.25 |  |  |  |  | 0.77 | 0.44 |
| **Model 3** |  |  | **0.060** | **0.033** | **2.25** | **6, 212** |  | **0.04** |
| Education | 0.10 | 0.26 |  |  |  |  | 0.39 | 0.70 |
| WTAR (SS) | 0.00 | 0.01 |  |  |  |  | 0.14 | 0.89 |
| **Race** | **-4.36** | **1.55** |  |  |  |  | **-2.81** | **0.005** |
| Model 4 |  |  | 0.062 | 0.031 | 1.99 | 7, 211 |  | 0.06 |
| SuperAger Status*Sex | 1.80 | 2.64 |  |  |  |  | 0.68 | 0.50 |

*Note.* *N*=226. SuperAger status was coded as 1=SuperAger, 0=Cognitively Average Control. WTAR: Wechsler Test of Adult Reading, SS: standardized score, adj.: adjusted. Race was coded as White (0) or non-White (1). Sex was coded as male (0) or female (1). SuperAger Status*Sex: interaction of SuperAger status and self-reported sex. Continuous predictors centered at their median: age 84, education 18 and WTAR 118. **Bolded models and predictors** indicate statistical significance.

**Supplemental Table 9. Emotional Support Regression.**

|  | B | SE (B) | R square | R square (adj.) | F | df1, df2 | t | *p* |
| --- | --- | --- | --- | --- | --- | --- | --- | --- |
| **Model 1** |  |  | **0.027** | **0.018** | **3.05** | **2, 223** |  | **0.049** |
| **Age** | **-0.32** | **0.13** |  |  |  |  | **-2.45** | **0.015** |
| Sex | -0.55 | 1.12 |  |  |  |  | -0.49 | 0.63 |
| Model 2 |  |  | 0.027 | 0.014 | 2.05 | 3, 222 |  | 0.11 |
| SuperAger Status | -0.28 | 1.15 |  |  |  |  | -0.24 | 0.81 |
| Model 3 |  |  | 0.037 | 0.010 | 1.36 | 6, 212 |  | 0.23 |
| Education | 0.20 | 0.25 |  |  |  |  | 0.79 | 0.43 |
| WTAR (SS) | -0.01 | 0.01 |  |  |  |  | -1.47 | 0.14 |
| Race | -0.26 | 1.45 |  |  |  |  | -0.18 | 0.86 |
| Model 4 |  |  | 0.039 | 0.007 | 1.22 | 7, 211 |  | 0.30 |
| SuperAger Status*Sex | 1.49 | 2.47 |  |  |  |  | 0.61 | 0.55 |

*Note.* *N*=226. SuperAger status was coded as 1=SuperAger, 0=Cognitively Average Control. WTAR: Wechsler Test of Adult Reading, SS: standardized score, adj.: adjusted. Race was coded as White (0) or non-White (1). Sex was coded as male (0) or female (1). SuperAger Status*Sex: interaction of SuperAger status and self-reported sex. Continuous predictors centered at their median: age 84, education 18 and WTAR 118. **Bolded models and predictors** indicate statistical significance.

**Supplemental Table 10. Perceived Stress Regression.**

|  | B | SE (B) | R square | R square (adj.) | F | df1, df2 | t | *p* |
| --- | --- | --- | --- | --- | --- | --- | --- | --- |
| Model 1 |  |  | 0.009 | 0.000 | 1.04 | 2, 223 |  | 0.36 |
| Age | 0.16 | 0.14 |  |  |  |  | 1.19 | 0.24 |
| Sex | 1.06 | 1.18 |  |  |  |  | 0.89 | 0.37 |
| Model 2 |  |  | 0.016 | 0.002 | 1.18 | 3, 222 |  | 0.32 |
| SuperAger Status | -1.44 | 1.20 |  |  |  |  | -1.20 | 0.23 |
| Model 3 |  |  | 0.017 | -0.011 | 0.59 | 6, 212 |  | 0.74 |
| Education | -0.05 | 0.26 |  |  |  |  | -0.21 | 0.84 |
| WTAR (SS) | -0.01 | 0.01 |  |  |  |  | -0.52 | 0.60 |
| Race | 0.99 | 1.53 |  |  |  |  | 0.65 | 0.52 |
| Model 4 |  |  | 0.017 | -0.016 | 0.51 | 7, 211 |  | 0.83 |
| SuperAger Status*Sex | 0.01 | 2.60 |  |  |  |  | 0.00 | 0.99 |

*Note.* *N*=226. SuperAger status was coded as 1=SuperAger, 0=Cognitively Average Control. WTAR: Wechsler Test of Adult Reading, SS: standardized score, adj.: adjusted. Race was coded as White (0) or non-White (1). Sex was coded as male (0) or female (1). SuperAger Status*Sex: interaction of SuperAger status and self-reported sex. Continuous predictors centered at their median: age 84, education 18 and WTAR 118. **Bolded models and predictors** indicate statistical significance.
